# Nitrous Oxide-Induced Cerebral Venous Thrombosis: A Case Report, Potential Mechanisms, and Literature Review

**DOI:** 10.7759/cureus.41428

**Published:** 2023-07-05

**Authors:** Athiphat Banjongjit, Panee Sutamnartpong, Piyanut Mahanupap, Pariya Phanachet, Sitthep Thanakitcharu

**Affiliations:** 1 Nephrology, Vichaiyut Hospital, Bangkok, THA; 2 Neurology, Vichaiyut Hospital, Bangkok, THA; 3 Hematology, Vichaiyut Hospital, Bangkok, THA; 4 Nutrition and Biochemical Medicine, Ramathibodi Hospital, Mahidol University, Bangkok, THA; 5 Pulmonology, Vichaiyut Hospital, Bangkok, THA

**Keywords:** cobalamin, vitamin b12, homocysteine, cerebral venous thrombosis, nitrous oxide

## Abstract

Cerebral venous thrombosis can result from hypercoagulation, either genetic or acquired. Hyperhomocysteninemia was previously thought to be linked with thrombophilia, although this is still controversial to this present day. In recent years, there has been a notable surge in the recreational use of nitrous oxide, which could potentially lead to hyperhomocysteinemia. We present a case of a 19-year-old female who was diagnosed with cerebral venous thrombosis with intracerebral hemorrhage. She had a history of nitrous oxide abuse, which is known to cause dysfunction of vitamin B12. Additionally, we conducted a literature review of cerebral venous thrombosis following nitrous oxide usage. Investigation showed that her serum vitamin B12 level was <100 pg/mL (reference range 197-771 pg/mL), and homocysteine level was 100.6 µmol/L (reference range 5.0-15.0 µmol/L). After receiving a vitamin B12 supplement, both serum vitamin B12 and homocysteine levels returned to normal. No other risk factors for thrombophilia were detected. Previously reported cases predominantly demonstrated hyperhomocysteinemia. The most likely mechanism of her cerebral venous thrombosis was hyperhomocysteinemia due to vitamin B12 deficiency caused by nitrous oxide abuse. This finding supports the hypothesis that hyperhomocysteinemia can induce cerebral venous thrombosis.

## Introduction

Cerebral venous thrombosis is one of the causes of stroke in young patients and can result in ischemic stroke, hemorrhagic transformation, and intraparenchymal hemorrhage. The most significant causes of cerebral venous thrombosis include oral contraceptive pills containing estrogen and hypercoagulable states [1]. Hyperhomocysteinemia is a rare cause of cerebral venous thrombosis [1]. However, some studies have shown that hyperhomocysteinemia does not increase the risk of venous thrombosis after adjusting for confounding factors [2-4]. Cobalamin deficiency can lead to hyperhomocysteinemia [5]. Nitrous oxide, also known as laughing gas, has seen an increase in recreational use in recent years. It can interfere with cobalamin function and cause hyperhomocysteinemia due to cobalamin deficiency [6]. Here, we report a case of a woman with cerebral venous thrombosis with both cobalamin deficiency and significant hyperhomocysteinemia resulting from nitrous oxide abuse.

## Case presentation

**Figure 1:**
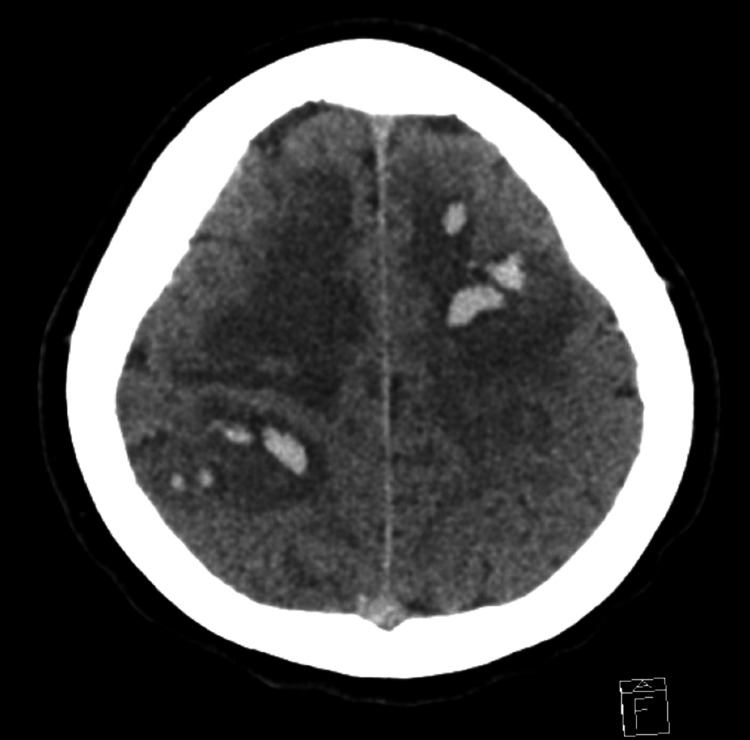
Several intraparenchymal hematomas involving the left frontal, right parietal, and right parietotemporal lobes, with perilesional edema

**Figure 2:**
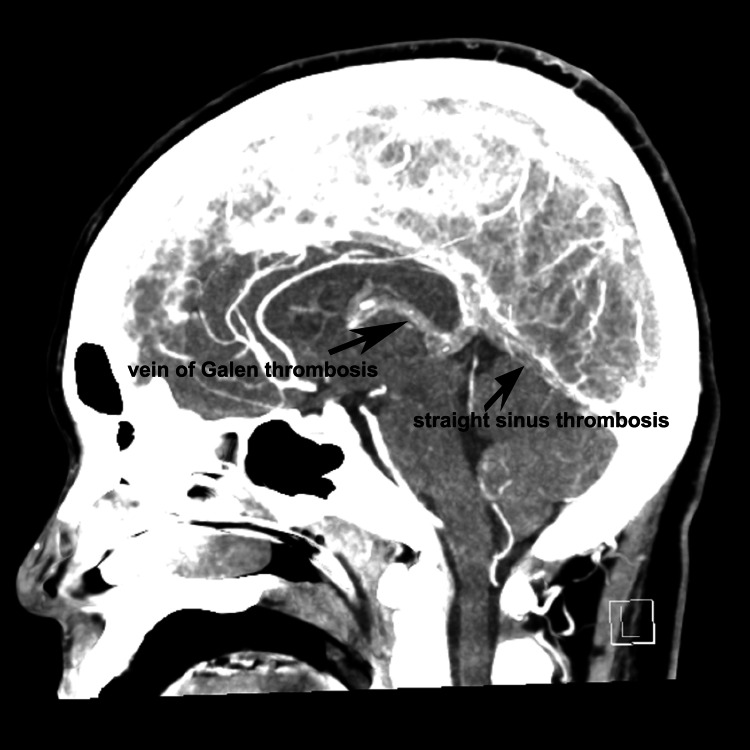
Thrombosis of internal cerebral veins, the vein of Galen, and straight sinus

**Figure 3:**
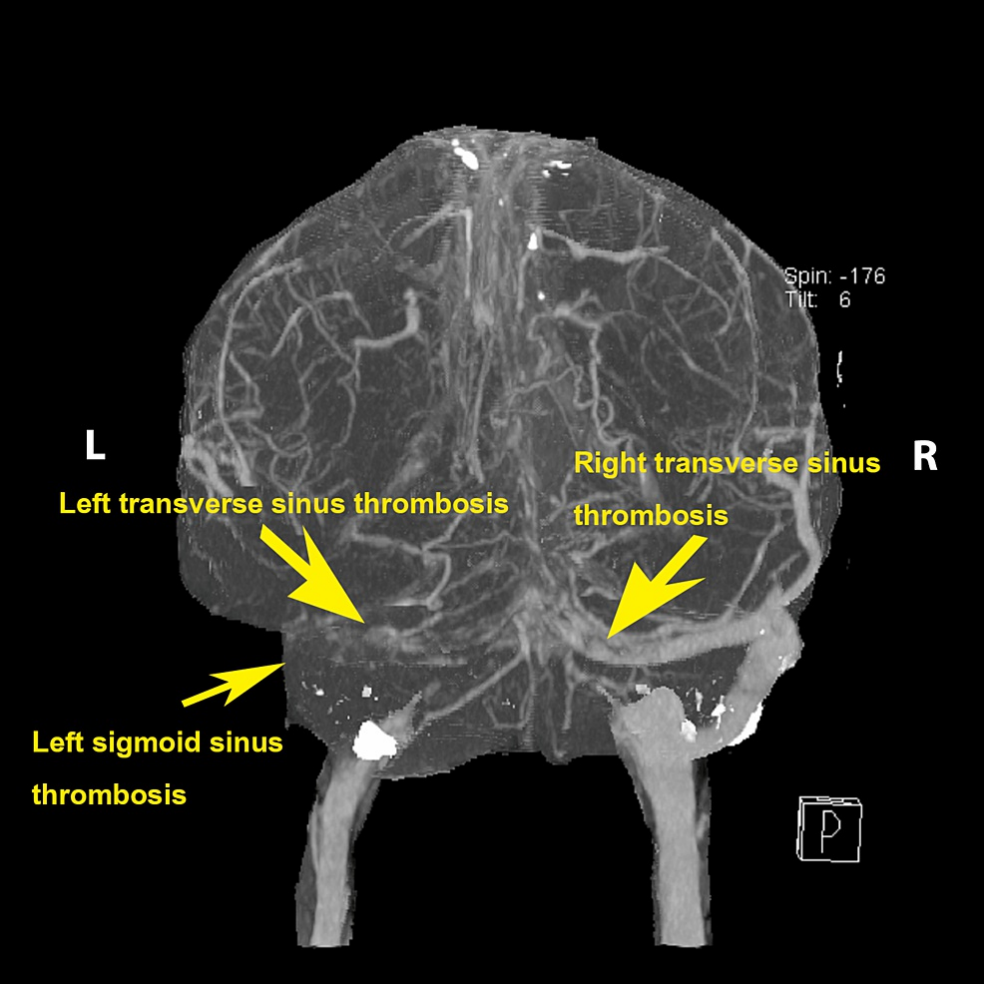
Thrombosis of bilateral transverse sinus and left sigmoid sinus

A 19-year-old female presented to the hospital with a three-day history of dizziness. She had no significant medical history but reported recreational use of nitrous oxide at a dosage of 320 grams per day for six months, and at a higher dosage of 2,880 grams per day for one month prior to admission, without using any other recreational drugs. She denied using oral contraceptive pills. She denied smoking and drinking alcohol. She is not a vegetarian and had a normal appetite. There were no abnormalities on the neurologic examination. Initial investigations revealed mild normocytic anemia (hemoglobin 10.5 g/dL, mean corpuscular volume (MCV) 91.6 fL, red cell distribution width (RDW) 13.5%, WBC 6,780/µL, and platelet 177,000/µL). After four hours of hospitalization, she developed a generalized clonic tonic seizure, and diazepam and levetiracetam were administered along with an endotracheal tube due to a stupor (E1V1M1). Computerized tomography (CT) of the brain with contrast showed several intraparenchymal hematomas (Figure 1). The CT cerebral venography (CTV) revealed extensive acute intraluminal thrombus in the superior sagittal sinus, bilateral internal cerebral veins, the vein of Galen, straight sinus, sinus confluence, bilateral transverse sinuses, the left sigmoid sinus to the left upper internal jugular vein, and cortical veins at bilateral high frontoparietal regions (Figures 2-3). The diagnosis of cerebral venous thrombosis resulting in intracerebral hemorrhage was made.

Intravenous unfractionated heparin (UFH) was administered and adjusted to maintain therapeutic range, with an activated partial thromboplastin time (aPTT) ratio (patient/control) of 1.5 to 2.5 for one week, and then switched to subcutaneous bemiparin (low-molecular-weight heparin (LMWH)). The seizure was controlled with lamotrigine and levetiracetam, and dexamethasone and mannitol were prescribed to reduce brain edema. Five days after the seizure, the patient’s consciousness was good, and she was extubated. The causes of venous thrombophilia were extensively investigated. According to the history of nitrous oxide usage, hyperhomocysteinemia from serum cobalamin deficiency was suspected. Investigations showed a significantly decreased serum cobalamin level (<100 pg/mL; reference range 197-771 pg/mL) and a significantly increased serum homocysteine level (100.6 µmol/L; reference range 5.0-15.0 µmol/L), supporting the diagnosis of thrombosis from hyperhomocysteinemia. Antiphospholipid antibodies (lupus anticoagulant, anti-beta-2 glycoprotein 1 IgM and IgG, anti-cardiolipin IgM and IgG) were negative. Serum protein C, protein S, and antithrombin III activity were normal (Table 1). 

**Table 1:** Laboratory investigation at admission MCV: Mean corpuscular volume

Parameters, Unit	Reference range	
Complete blood count		
Hemoglobin, g/dL	10.5	11.5-16.5
MCV, fL	91.6	80.0-95.0
WBC, cells/µL	6,780	4,500-11,000
Platelet, cells/µL	177,000	140,000-400,000
Serum cobalamin level, pg/mL	<100	197-771 pg/mL
Serum homocysteine level, µmmol/L	100.6	5.0-15.0
Lupus anticoagulant	Negative	Negative
Anti beta-2 glycoprotein 1 IgM	Negative	Negative
Anti beta-2 glycoprotein 1 IgG	Negative	Negative
Anti-cardiolipin IgM	Negative	Negative
Anti-cardiolipin IgG	Negative	Negative
Serum protein C activity, %	99.3	64.0-141.0
Serum protein S activity, %	115.1	61.0-127.0
Serum antithrombin III activity, %	93.7	80.0-128.0

As the patient was receiving anticoagulant therapy, 1000 µg of vitamin B12 was subcutaneously injected daily to prevent bleeding from the intramuscular injection. Seven days after daily subcutaneous vitamin B12 administration, serum vitamin B12 and homocysteine levels were measured with results of >2000 pg/mL (reference range 197-771 pg/mL) and 11.1 µmol/L (reference range 5.0-15.0 µmol/L), respectively.

The patient was hospitalized for one month, during which time her consciousness returned to normal and her motor power gradually improved to the point where she could perform basic activities of daily living. All muscle motor power was grade V except for left foot dorsiflexion, which was grade III. Due to the delayed recovery of peripheral weakness and numbness, a nerve conduction study (NCS) test was performed. The test revealed mainly axonal polyneuropathy with mixed motor and sensory fibers. She was discharged with a plan for warfarin therapy for three months as per provoked venous thromboembolism. Vitamin B12 1000 µg was continued to be subcutaneously injected weekly for one month.

## Discussion

We present a case of extensive cerebral venous thrombosis with intracerebral hemorrhage in a young female with a history of nitrous oxide abuse. Investigations revealed a significantly decreased serum cobalamin level and an elevated serum homocysteine level. After cobalamin supplementation, the homocysteine level returned to normal. Other thrombophilic factors were not detected, indicating that hyperhomocysteinemia associated with nitrous oxide abuse was the likely cause of cerebral venous thrombosis. She also had axonal polyneuropathy, which was likely caused by cobalamin deficiency [7].

Thrombophilia screening is not recommended for patients with cerebral venous thrombosis by the European Academy of Neurology due to controversial evidence regarding its efficacy in reducing mortality, improving functional outcomes, or preventing recurrent venous thrombosis. Nevertheless, it is important to inquire about a history of estrogen-containing oral contraceptive pill use, which is strongly associated with a 7.6-fold increase in the risk of cerebral venous thrombosis [8]. The decision to conduct thrombophilia screening should be individualized, as in our case, where the patient had a history of nitrous oxide abuse that has been associated with an increased risk of cerebral venous thrombosis. As a result, the serum level of homocysteine was measured, revealing hyperhomocysteinemia.

**Figure 4:**
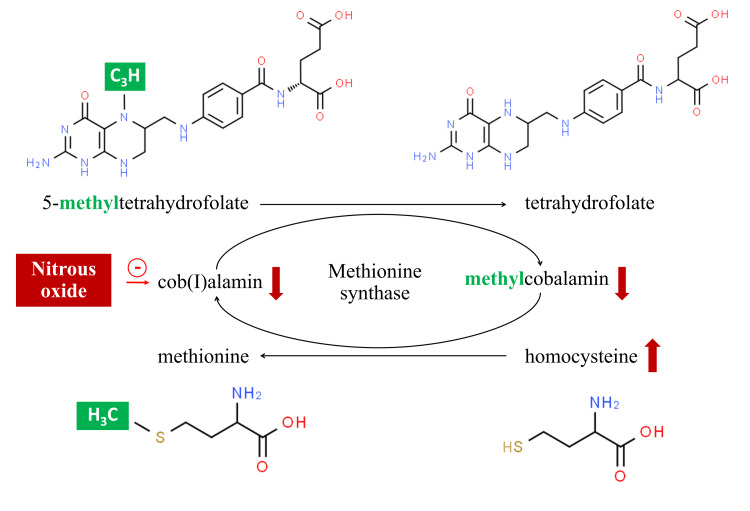
Cobalamin, in the form of cob(I)alamin, carries a methyl group from 5-methyltetrahydrofolate and transfers it to homocysteine in the form of methylcobalamin. This methionine cycle is affected by nitrous oxide, which reduces cob(I)alamin and methylcobalamin, leading to an increase in homocysteine levels. This image was created by the authors using Microsoft PowerPoint (Microsoft Corp., Redmond, WA, USA).

Homocysteine is a factor in the methionine cycle that is associated with cobalamin, a common natural coenzyme. The enzyme methionine synthase (MS) utilizes methylcobalamin and 5-methyltetrahydrofolate as cofactors to facilitate the conversion of homocysteine to methionine through remethylation. When cobalamin attaches to MS, it is reduced to cob(I)alamin, the most reactive form of cobalamin, which transfers a methyl group from 5-methyltetrahydrofolate, forming methylcobalamin and tetrahydrofolate. Methylcobalamin then transfers the methyl group to homocysteine, resulting in the production of methionine [5]. Nitrous oxide can convert cob(I)alamin to cob(II)alamin, which is unable to carry methyl groups. This prevents the conversion of homocysteine to methionine, leading to hyperhomocysteinemia (Figure 4) [6]. Nitrous oxide abuse has been reported to be associated with thrombotic events, including ischemic stroke, acute coronary syndrome, and venous thromboembolism [9-14]. To our knowledge, there are six previous case reports of suspected nitrous oxide-induced cerebral venous thrombosis in which baseline serum homocysteine and cobalamin levels were presented (Table 2), and only two patients had cobalamin deficiency [10,11]. However, it should be noted that patients with serum cobalamin levels within the gray area of 170-340 pg/mL could have cobalamin deficiency and should be tested for plasma methylmalonic acid or homocysteine levels, which are more sensitive [15]. The complete blood count (CBC) could show no evidence of megaloblastic anemia or pancytopenia despite cobalamin deficiency, as was also observed in our case. Our patient had mixed motor and sensory fibers, which was due to cobalamin deficiency. Due to varying reports of dosage and duration, it is challenging to interpret the correlation between the amount and length of nitrous oxide usage and the severity of its effects (Table 2).

**Table 2:** Previous reports of nitrous oxide-induced cerebral venous thrombosis MTHFR: Methylenetetrahydrofolate reductase

First author, year	Country	Age (years)	Sex and distinctive characteristics	Dosage of nitrous oxide	Brain imaging	Homocysteine (µmol/L)*	Cobalamin (pg/mL)**
Lui M [12], 2020	China	25	Female on oral contraceptive pills	Intermittently for 20 months	Isolated cortical vein thrombosis with hemorrhagic infarction and subarachnoid hemorrhage involving the left parietal lobe	13.6 µmol/L	Normal
Pratt DN [13], 2020	USA	21	Female with 11 weeks pregnancy and homozygous MTHFR polymorphism	4,800 grams/day for several weeks	The left transverse sinus, sigmoid sinus, and internal jugular vein thrombosis	> 65 μmol/L	Normal
Lin SS [10], 2022	Taiwan	25	Male	Not mentioned	The straight sinus and bilateral transverse sinuses thrombosis	59.03 μmol/L	<148 pg/mL
Farhat W [11], 2022	France	16	Female	Up to 144 grams/day for 3 weeks	Superior sagittal sinus, straight sinus, transverse sinuses, right sigmoid sinus, right jugular vein, and superficial and deep cerebral veins thrombosis	134 μmol/L	93 pg/mL
Peng C [14], 2023	China	20	Male	Unclear dosage for 3 months	Superior sagittal sinus, left transverse sinus, and left sigmoid sinus thrombosis and the left temporal lobe infarction with hemorrhage	26.3 μmol/L	243 pg/mL
Caris MG [9], 2023	Netherlands	18	Female	2,000 grams every 2 weeks (sharing with friends)	The left jugular vein to the sigmoid and transverse sinuses thrombosis with hemorrhagic infarction in the left parietal and occipital lobes	67 μmol/L	237 pg/mL
Banjongjit A, 2023 (this case)	Thailand	19	Female	320 grams/day for 6 months, and 2,880 grams/day for one month	Superior sagittal sinus, bilateral internal cerebral veins, the vein of Galen, straight sinus, sinus confluence, bilateral transverse sinuses, the left sigmoid sinus to the left upper internal jugular vein, and cortical veins at bilateral high frontoparietal regions thrombosis with hematomas involving the left frontal, right parietal, and right parietotemporal lobes, with perilesional edema	100.6 µmol/L	< 100 pg/mL
* Serum homocysteine level: reference range 5.0-15.0 µmol/L **Serum cobalamin level: reference range 197-771 pg/mL							

Although the linkage between hyperhomocysteinemia and thrombophilia is unclear [2-4], it could be postulated that elevated serum homocysteinemia levels could lead to cerebral venous thrombosis among patients who abuse nitrous oxide, as demonstrated by our case and other previous reports. This is also supported by the mechanistic effects of nitrous oxide on cobalamin and homocysteine.

## Conclusions

In conclusion, nitrous oxide abuse could lead to cerebral venous thrombosis through hyperhomocysteinemia caused by B12 deficiency. Thus, the treatment is B12 supplementation. Further research is needed to establish a definitive confirmation of the link between hyperhomocysteinemia and thrombophilia. Clinicians should keep in mind the association between nitrous oxide abuse and venous thrombotic events as well as other neurological conditions, including peripheral neuropathy. It is important to inquire about the history of substance use when suspecting cerebral venous thrombosis, especially in the younger generation.
